# Hydrogen-Bond Organization and Porous Architecture Govern Water Transport and Germination in Cellulosic Membranes

**DOI:** 10.3390/polym18131575

**Published:** 2026-06-24

**Authors:** Natalia Fuentes Molina, Ana Fragozo Molina, Kennys Cujia Jiménez

**Affiliations:** Members of the Research Group Semiarid Territory of the Caribbean, Environmental Engineering, Faculty of Engineering, Universidad de La Guajira, Riohacha 440003, Colombia; acfragozo@uniguajira.edu.co (A.F.M.); kdcujia@uniguajira.edu.co (K.C.J.)

**Keywords:** porous cellulose membranes, lignocellulosic biomass, hydrogen bonding, water vapor transmittance, biodegradable mulch, nature-based solutions, semi-arid agriculture, seed germination

## Abstract

Water scarcity in semi-arid regions threatens seed germination and early crop establishment, driving the development of biodegradable Nature-based Solutions to replace synthetic plastic mulches. Porous cellulose membranes were fabricated from rice husk (RH), banana pseudostem (BP), and sugarcane bagasse (SB) by thermo-chemical extraction and high-shear homogenization (*n* = 5 replicates per membrane type). Membranes were characterized by ATR-FTIR and scanning electron microscopy, confirming removal of non-cellulosic components and biogenic silica preservation in RH, and revealing biomass-dependent porous architectures linked to mechanical and transport behavior. RH produced the most compact fibrillar matrix (compressive strength: 8.16 ± 0.24 MPa; WVT: 170 ± 60 g m^−2^ day^−1^), BP an open interconnected network with superior deformability (9.83 ± 0.25% elongation) and moisture transport (WVT: 400 ± 100 g m^−2^ day^−1^), and SB the highest moisture-retention capacity (215.7 ± 15.8%). Germination assays with *Brassica oleracea* var. *botrytis* under water stress showed SB achieved the highest germination rate (90.5 ± 0.99%), confirming that sustained moisture availability governs germination more decisively than transport rate alone. Soil burial tests confirmed biodegradable behavior across all membranes (R^2^ ≥ 0.995; k = 0.043–0.046 day^−1^). These findings establish a hydrogen-bond-mediated structure–property–function framework for designing biomass-specific cellulose membranes as biodegradable solutions for water-limited agricultural systems.

## 1. Introduction

Global food security is increasingly threatened by climate change, particularly in semi-arid regions where erratic rainfall and high evapotranspiration rates impair seed germination and early seedling establishment [1,2,3]. In territories such as La Guajira, Colombia, persistent water deficit during early phenological stages frequently compromises crop productivity. Low-density polyethylene (LDPE) mulch films are the dominant agronomic response, currently covering more than 30 million hectares of agricultural land worldwide [4,5,6]; however, their resistance to biodegradation (~100 years under natural soil conditions) drives the accumulation of an estimated 1.5–6.6 million tons of microplastic residues in global agricultural soils [6,7], altering soil structure, microbial activity, and water-holding capacity. This environmental burden motivates the development of biodegradable, bio-based alternatives capable of matching the moisture-conservation performance of polyolefin films [4,5,6,7,8].

Cellulose membranes derived from agro-industrial residues have emerged as promising Nature-based Solutions (NbS) for sustainable moisture management. Recent advances involving sugarcane bagasse nanofibrils [9] and banana pseudostem-derived bioplastics [10] demonstrate the feasibility of converting agricultural residues into functional materials with tunable structural and transport properties [10,11,12,13,14]. However, a critical challenge persists in balancing mechanical resistance and moisture transport. At the nanoscale, ambient humidity disrupts inter-chain hydrogen bonds within cellulose nanofibers, reducing network cohesion and triggering plasticization that progressively lowers stiffness with rising relative humidity [15,16]. At the macroscopic scale, this translates into reduced tensile strength and altered water vapor transmittance, directly affecting the functional performance of cellulose-based membranes under field humidity gradients [17]. This humidity-driven stiffness–permeability trade-off is decisive in agricultural mulch applications: high crystallinity confers field durability [17,18,19] but restricts the moisture accessibility required for seed imbibition under water stress [20,21,22], necessitating systematic evaluation of moisture transport under controlled humidity gradients.

At the molecular level, membrane functionality is governed by the spatial organization of cellulose chains and the density of intra- and intermolecular hydrogen-bond networks. Surface hydroxyl (–OH) groups promote self-assembly during solvent evaporation, generating hierarchical porous architectures that simultaneously regulate structural cohesion and water–matrix interactions [14,23]. Under field humidity gradients, water molecules occupy amorphous inter-fibrillar regions, competing with and displacing inter-chain hydrogen bonds, thereby reducing nanoscale network cohesion and coupling the mechanical state of the membrane to its vapor transport capacity [24,25]. Biomass origin modulates this humidity-driven response through differences in mineral composition, cellulose accessibility, and inorganic domain distribution, altering surface energy, hydrogen-bond density, and the sorption isotherm under the 50–90% RH gradients characteristic of semi-arid agricultural environments [14,23]. These structural features ultimately determine the capacity of cellulose matrices to regulate transitions between bound and mobile water, governing the localized moisture availability critical for germinating seeds under hydric stress [20,26].

The behavior of cellulose-based membranes is also strongly influenced by the intrinsic chemical composition of the precursor biomass. Agro-industrial residues differ substantially in cellulose, hemicellulose, lignin, ash, and mineral content, factors that directly affect fibrillation efficiency, hydroxyl accessibility, and supramolecular organization during membrane formation [12,13]. Rice husk is characterized by a high silica and ash fraction that may influence interfacial interactions and promote compact fibrillar organization, whereas banana pseudostem contains lower inorganic content and more flexible lignocellulosic domains that favor fibrillar separation and porous assembly [10,17]. Sugarcane bagasse represents an intermediate system rich in structural carbohydrates and residual lignin fractions, frequently associated with balanced mechanical and transport behavior [12,13]. Consequently, biomass origin should not be interpreted merely as a raw material source but as a compositional variable capable of governing hydrogen-bond organization, water transport dynamics, and biological functionality in cellulose-derived materials.

Water transport in cellulose membranes is governed by coupled sorption and diffusion processes strongly influenced by fibrillar organization, pore connectivity, and the relative distribution of amorphous and crystalline domains [14,17]. More compact and crystalline structures may provide greater dimensional stability and lower permeability, whereas more open and less densely packed architectures tend to enhance hydrophilicity, moisture accessibility, and structural deformability [22,23]. Consequently, the balance between hydrogen-bond-mediated cohesion and water affinity emerges as a key design principle for developing cellulose membranes capable of regulating moisture dynamics under drought-prone conditions [12,27].

Despite this progress, no systematic study has directly compared how compositionally contrasting lignocellulosic sources under identical extraction and membrane fabrication conditions produce differentiated hydrogen-bond networks and porous architectures, and whether these microstructural differences translate into functionally relevant outcomes for seed germination and early plant establishment under hydric stress. We hypothesize that biomass-specific chemical composition, particularly differences in silica content, lignocellulosic recalcitrance, and cellulose accessibility, generates distinct hydrogen-bond organization and pore connectivity during membrane consolidation, which in turn governs moisture transport capacity, mechanical behavior, and agronomic performance under water-limited conditions. To test this hypothesis, this study fabricates and comparatively evaluates porous cellulose membranes from rice husk (RH), banana pseudostem (BP), and sugarcane bagasse (SB) through a standardized thermo-chemical extraction and high-shear homogenization protocol, assessing membrane microstructure (FTIR, SEM), mechanical and barrier properties (compressive strength, elongation, water vapor transmittance), and functional agronomic performance (water retention, seed germination, and biodegradation) within a single integrated platform. The findings aim to provide a rational basis for the biomass-specific design of biodegradable cellulose membranes as Nature-based Solutions for moisture regulation in water-limited agricultural systems.

## 2. Materials and Methods

### 2.1. Raw Materials and Selective Cellulose Extraction

Lignocellulosic residues including rice husk (RH) (*Oryza sativa* L.), banana pseudostem (BP) (*Musa* sp.), and sugarcane bagasse (SB) (*Saccharum officinarum* L.) were collected from agricultural zones of the Colombian Caribbean region. The raw materials were washed with distilled water and subjected to controlled boiling at 90 °C for 1 h to remove soluble extractives and low-molecular-weight compounds. The materials were subsequently dried at 40 °C for 24 h and milled to particle sizes ranging from 70 to 150 µm to improve surface area and chemical accessibility [1,13].

Cellulose extraction was carried out through a selective thermo-chemical process. Alkaline delignification was performed using 5 wt.% NaOH at a solid-to-liquid ratio of 1:20 (*w*/*v*) and 80 °C for 4 h under continuous stirring to promote lignin and hemicellulose removal while minimizing excessive mineral dissolution in RH [13,28]. After washing to neutral pH, oxidative bleaching was conducted using 3 wt.% H_2_O_2_ in alkaline medium at 70 °C for 1 h to eliminate residual chromophoric compounds and remaining lignin fractions [29]. A complementary purification step using aqueous ethanol (70% *v*/*v*) was subsequently applied to remove residual extractives. The purified fibers were then conditioned with 1% (*v*/*v*) acetic acid to promote mild surface activation and increase the accessibility of hydroxyl groups, thereby enhancing intermolecular hydrogen bonding and water–matrix interactions in the resulting membranes [13,17,23].

### 2.2. Preparation of Porous Cellulose Membranes

Cellulose suspensions were prepared by dispersing purified fibers in distilled water at 2 wt.% and mechanically homogenizing them using a high-shear rotor–stator system (15,000 rpm, 10 min). Homogenization was conducted under ice-bath cooling to maintain temperatures below 30 °C and minimize thermal hornification while preserving fibrillar organization and pore-forming capacity [13]. Glycerol (10 wt.% relative to dry cellulose mass) was incorporated as a plasticizer prior to casting. Subsequently, 40 mL of suspension was cast onto leveled glass plates and dried at 40 °C for 48 h, producing membranes with thicknesses ranging from 94 to 96 µm [17].

### 2.3. Structural, Mechanical and Barrier Characterization

Surface morphology and pore interconnectivity were examined by scanning electron microscopy (ZEISS EVO 15(Carl Zeiss Microscopy GmbH, Jena, TH, Germany) y JEOL JSM-5910LV(JEOL Ltd., Akishima, Tokyo, Japan) operated at an accelerating voltage of 10 kV after sputter-coating specimens with a ~10 nm gold layer to ensure electrical conductivity. Micrographs were acquired at magnifications of 500× and 2000× for each membrane type. Functional groups and hydrogen-bond-associated spectral features were evaluated by attenuated total reflectance Fourier-transform infrared spectroscopy (ATR-FTIR; Perkin-Elmer Frontier, Waltham, MA, USA) over the 4000–500 cm^−1^ range, with a spectral resolution of 4 cm^−1^ and 32 co-added scans per spectrum. Three independent spectra were collected per membrane type to verify reproducibility [18,30]. Mechanical characterization was conducted using a universal testing machine (*n* = 5 specimens per membrane type) at 25 °C and 50% relative humidity (RH). Elongation at break (*ε_b_*, %) was determined according to ASTM D882 and calculated as:

(1) $$\varepsilon_{b}=\frac{L_{f}-L_{0}}{L_{0}}\times 100$$

where *L_f_* is the gauge length at fracture (mm) and *L*_0_ is the initial gauge length (mm). Compressive strength (*σ_c_*, MPa) was evaluated following ASTM D695 at a crosshead speed of 1.3 mm min^−1^ and calculated as:

(2) $$\sigma_{c}=\frac{F}{A}$$

where *F* is the maximum compressive force applied (N) and *A* is the original cross-sectional area of the specimen (mm^2^) [18,27]. Water Vapor Transmittance (WVT, g m^−2^ day^−1^) was determined gravimetrically following the desiccant method (ASTM E96, Procedure A). Membrane specimens were sealed over cylindrical permeation cells containing anhydrous silica gel as desiccant, with an effective exposed area (*A*) of 10 cm^2^. Cells were maintained at 25 °C under a relative humidity gradient of 50–90% RH for a period of 24 h. Mass changes (Δ*m*, g) were recorded at 2 h intervals to verify attainment of steady-state transport conditions, and *WVT* was calculated as:

(3) $$WVT=\frac{\Delta m}{A\times t}$$

where Δ*m* is the mass change in the permeation cell (g) recorded at steady state, *A* is the effective exposure area (m^2^), and *t* is the transmission time (day). All *WVT* measurements were performed in triplicate (*n* = 3) [17,31].

### 2.4. Functional Performance Water Retention, Germination and Biodegradation

Functional performance was evaluated using five replicates per treatment (*n* = 5). Water-retention capacity was assessed using 200 g of agricultural soil covered with membrane samples and monitored every 48 h over a 10-day period to simulate moisture conservation under semi-arid conditions [26]. Germination assays were conducted using *Brassica oleracea* var. *botrytis* under controlled water-stress conditions. Germination percentage, shoot length, and root length were recorded to evaluate early plant vigor and membrane-assisted biological performance [20,22,26]. Biodegradation behavior was assessed through a controlled soil burial assay performed at 10 cm depth under 25 °C and 60% relative humidity (RH) over a 56-day period [17,25]. The selected burial depth was adopted because it represents an active soil zone characterized by stable soil–material interaction and relevant microbial activity, consistent with previous biodegradation studies of cellulose-based agricultural materials [23,31]. The soil used for degradation experiments exhibited a sandy loam texture, alkaline pH (7.8), and organic matter content of 4.5%, conditions that influence microbial colonization and cellulose depolymerization. Membrane mass loss was measured at 0, 7, 14, 28, and 56 days. Degradation kinetics were modeled using a first-order exponential equation to estimate the degradation constant (k) and half-life (t_50_), providing a quantitative assessment of membrane persistence and biodegradation dynamics [24,25,27].

Two additional control treatments were included: bare soil without membrane under the same water-stress conditions to establish baseline germination performance, and low-density polyethylene (LDPE) mulch film (25 µm thickness) under equivalent water-limited conditions to benchmark performance against the conventional plasticulture standard [8,9].

### 2.5. Statistical Analysis

Statistical analyses were performed using OriginPro 2024 software (OriginLab Corporation, Northampton, MA, USA). Experimental data obtained from five independent replicates (*n* = 5) are reported as mean ± standard deviation. Prior to statistical analysis, data normality and homogeneity of variance were assessed using the Shapiro–Wilk and Levene tests, respectively. Differences among membranes derived from distinct biomass sources were evaluated using one-way analysis of variance (ANOVA), followed by Tukey’s honestly significant difference (HSD) post hoc test for pairwise comparisons. Statistical significance was established at *p* < 0.05.

## 3. Results and Discussion

### 3.1. Structural and Chemical Characterization

**Figure 1:**
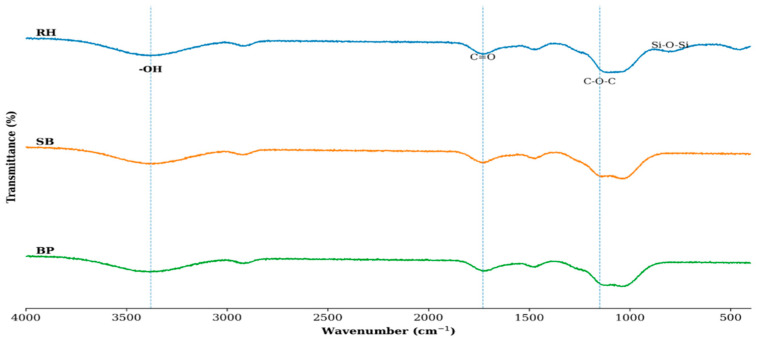
Fourier transform infrared (FTIR) spectra of porous cellulose membranes derived from rice husk (RH), sugarcane bagasse (SB), and banana pseudostem (BP), The vertical dashed lines indicate the characteristic absorption bands of the key functional groups (-OH, C=O, and COC).

The structural and chemical characteristics of the engineered cellulose membranes were evaluated through FTIR spectroscopy and scanning electron microscopy (SEM). FTIR spectra (Figure 1) confirmed successful cellulose isolation across all biomass sources. The disappearance of absorption bands at approximately 1730 cm^−1^ (C=O stretching of hemicellulose acetyl groups) and 1510 cm^−1^ (aromatic C=C stretching of lignin) indicated effective removal of non-cellulosic components following thermo-chemical treatment. The presence of characteristic cellulose bands—including C–H stretching near 2900 cm^−1^ and C–O–C glycosidic stretching near 1050 cm^−1^—confirmed the integrity of the cellulosic backbone in all membranes.

All membranes exhibited a broad O–H stretching band centered near 3330 cm^−1^, associated with hydroxyl groups involved in intra- and intermolecular hydrogen bonding. The relative intensity of this band was more pronounced in RH membranes compared with BP and SB, suggesting differences in hydroxyl group density and hydrogen-bond organization among biomass sources. Additionally, RH membranes exhibited characteristic Si–O–Si vibration bands between 800 and 1100 cm^−1^, confirming the preservation of biogenic silica following thermo-chemical treatment, a feature not observed in BP or SB spectra.

**Figure 2:**
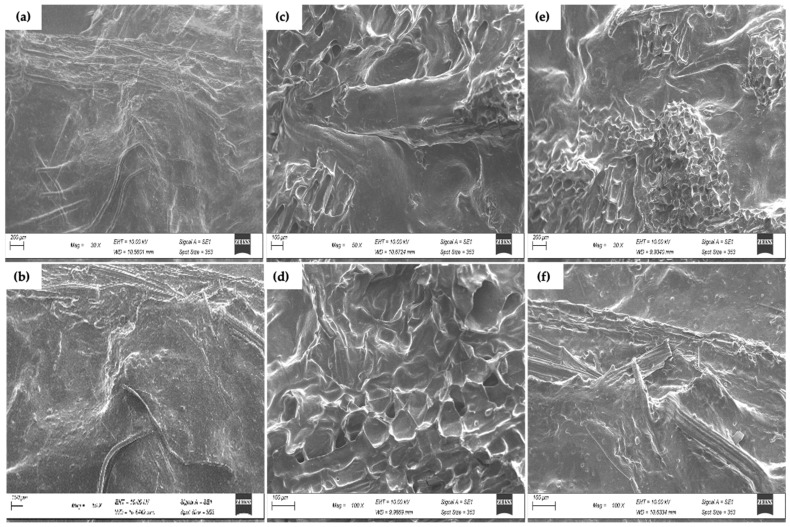
Scanning electron microscopy (SEM) micrographs of porous cellulose membranes derived from different lignocellulosic sources (**a**,**b**) rice husk (RH), (**c**,**d**) banana pseudostem (BP), and (**e**,**f**) sugarcane bagasse (SB) at 500× ((**left**) panels) and 2000× ((**right**) panels) magnification. Pore-size ranges reported in the text were estimated by systematic visual inspection across five independent specimens per membrane type at both magnifications.

Scanning electron microscopy (SEM) confirmed the formation of interconnected porous networks in all membranes, with distinct architectures depending on the biomass source (Figure 2). Pore-size ranges were estimated by systematic visual inspection of micrographs acquired at two magnifications (500× and 2000×) across five independent membrane specimens per biomass source, ensuring that the reported dimensions reflect consistent, reproducible structural features rather than isolated observations. RH membranes exhibited a compact fibrillar structure with reduced pore connectivity; pore sizes were consistently below 5 µm across all inspected images at both magnifications, with no evidence of larger voids or interconnected macropores (Figure 2a,b), a structural outcome attributed to silica-mediated confinement of cellulose chains during membrane consolidation. BP membranes displayed a highly open and interconnected network with larger and more irregular pores; diameters ranging from 10 to 30 µm were repeatedly identified at 500× magnification, and the heterogeneous pore geometry including elongated channels and irregular voids was confirmed at 2000× across all replicate specimens (Figure 2c,d), consistent with the lower mineral interference and greater fibrillar separability of banana pseudostem-derived cellulose. SB membranes presented an intermediate morphology, characterized by a heterogeneous distribution of dense fibrillar regions and microvoids; pore sizes in the range of 5–15 µm were consistently observed at both magnifications across all replicate specimens. These biomass-dependent differences in fibrillar packing density and pore geometry are consistent with the FTIR-identified variations in hydroxyl group accessibility and hydrogen-bond organization.

### 3.2. Physical, Mechanical and Barrier Properties

Physical characterization revealed biomass-dependent differences in membrane density and water interaction behavior (Table 1). RH membranes exhibited the highest density (1.33 ± 0.02 g cm^−3^), followed by BP (1.15 ± 0.06 g cm^−3^) and SB (0.89 ± 0.10 g cm^−3^), consistent with the compact fibrillar organization observed by SEM. Water absorption capacity followed the inverse trend, with SB achieving the highest value (485.3 ± 22.4%), followed by BP (311.8 ± 18.6%) and RH (186.4 ± 10.2%), reflecting differences in pore accessibility and available sorption sites among membranes (*p* < 0.05).

**Figure 3:**
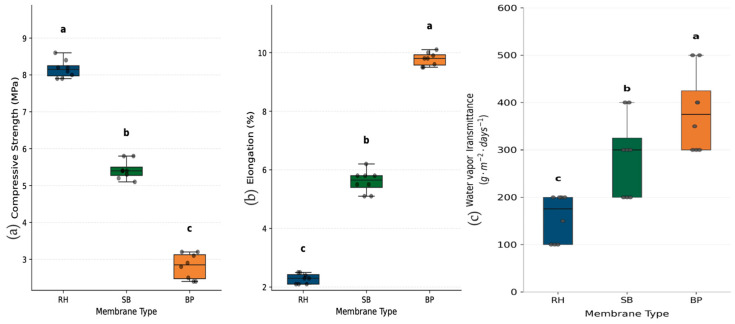
Mechanical and barrier properties of porous cellulose membranes. Boxplot representation of (**a**) compressive strength, (**b**) elongation, and (**c**) water vapor transmittance for membranes derived from RH, SB, and BP. Data are presented as median, interquartile range, and outliers, while individual points correspond to replicates of each measurement. Different letters indicate statistically significant differences (*p* < 0.05).

Compressive testing revealed significant biomass-dependent mechanical behavior (*n* = 5, *p* < 0.05) (Table 1, Figure 3). RH membranes exhibited the highest compressive strength (8.16 ± 0.24 MPa), followed by SB (5.39 ± 0.31 MPa) and BP (2.81 ± 0.15 MPa). Elongation at break showed an inverse trend: BP displayed the highest deformability (9.83 ± 0.25%), followed by SB (5.65 ± 0.47%) and RH (2.33 ± 0.17%). This stiffness–flexibility trade-off was statistically significant across all pairwise comparisons (*p* < 0.05), as indicated by distinct letters in Table 1.

Barrier properties followed the same biomass-dependent pattern (Table 1, Figure 3c). Water Vapor Transmittance (WVT) differed significantly among membranes (*p* < 0.05), increasing from RH (170 ± 60 g m^−2^ day^−1^) to SB (300 ± 100 g m^−2^ day^−1^) and BP (400 ± 100 g m^−2^ day^−1^), a range consistent with values reported for unmodified cellulose nanofibril and microfibril films where moisture transport is primarily governed by fibrillar packing density and free volume rather than by biomass chemical composition alone [32].

The functional behavior of the cellulose membranes originated from the interaction between thermo-chemical pretreatment and biomass-specific chemical composition, particularly the differential response of each lignocellulosic source to alkaline delignification and oxidative bleaching. Both treatments disrupted lignin–carbohydrate complexes and increased cellulose chain accessibility, exposing surface hydroxyl groups that subsequently drove intermolecular hydrogen bonding during membrane formation and solvent evaporation [28,29]. However, the structural outcome of this process was strongly governed by biomass recalcitrance and chemical heterogeneity, rather than by treatment conditions alone.

In RH, the preservation of Si–O–Si vibration bands between 800 and 1100 cm^−1^ confirmed that biogenic silica was not fully dissolved under the alkaline conditions applied. This is consistent with the known chemical resistance of amorphous silica in rice husk, which requires stronger conditions (e.g., higher NaOH concentrations or temperatures above 120 °C) for complete dissolution [19]. Rather than acting as passive residues, these silica-rich domains likely altered local surface energy, restricted fibrillar mobility during solvent evaporation, and promoted closer fibrillar packing, thereby increasing hydrogen-bond density and network cohesion. This mineral–cellulose co-organization is consistent with silica-reinforced cellulose composites reported in the literature, where inorganic domains enhance load-bearing capacity through confinement effects and interfacial stress transfer [19,23], and is reflected in the highest compressive strength observed for RH membranes (8.16 ± 0.24 MPa).

In contrast, BP presented the lowest mineral interference and greatest fibrillar separability among the evaluated sources. The absence of prominent inorganic domains facilitated more extensive fibrillation during high-shear homogenization and promoted an open, interconnected porous assembly during drying. This behavior aligns with reports on banana pseudostem-derived cellulose, where reduced inorganic content and inherently flexible fibrillar organization favor accessible hydrogen-bond networks and low-resistance pore formation [10,14], consistent with the lowest compressive strength (2.81 ± 0.15 MPa) and highest elongation (9.83 ± 0.25%) recorded for BP membranes in this study.

SB exhibited an intermediate response reflecting its transitional lignocellulosic composition, characterized by partial lignin and hemicellulose removal and moderate fibrillar cohesion. The resulting microstructure balanced fibrillar accessibility with structural integrity, producing membranes of intermediate mechanical performance (5.39 ± 0.31 MPa compressive strength; 5.65 ± 0.47% elongation). Collectively, these observations indicate that pretreatment effectiveness cannot be evaluated exclusively as a process parameter; it must be interpreted as a biomass-dependent interaction between cellulose accessibility, residual inorganic and lignocellulosic domains, and self-assembly dynamics during membrane consolidation [1,13,14,23].

### 3.3. Water Interaction and Retention Dynamics

Water absorption and retention capacity differed significantly among membranes (*p* < 0.05), reflecting biomass-dependent differences in pore accessibility and internal surface area (Table 1). SB membranes exhibited the highest water absorption (485.3 ± 22.4%) and retention capacity (215.7 ± 15.8%), followed by BP (absorption: 311.8 ± 18.6%; retention: 145.3 ± 12.3%) and RH (absorption: 186.4 ± 10.2%; retention: 92.1 ± 6.5%). All pairwise comparisons were statistically significant (*p* < 0.05).

Temporal monitoring of soil moisture retention over 10 days confirmed these trends under simulated semi-arid conditions. SB membranes maintained the highest moisture content throughout the evaluation period, showing a gradual and sustained decline from the initial value, with retention remaining above 60% of the initial water content at day 10. BP membranes exhibited a more rapid initial decline in moisture content during the first 48–96 h, stabilizing at intermediate values thereafter. RH membranes showed the steepest moisture loss curve, reaching the lowest retention values by day 6 and approaching baseline levels by day 10. These temporal profiles confirm that the three biomass sources produce functionally distinct moisture regulation behaviors under conditions representative of water-limited agricultural environments.

Membrane functionality under water-limited conditions was governed by the spatial organization of the porous network and the chemical accessibility of hydroxyl-rich sorption sites, rather than by total porosity alone. Water transport in cellulose matrices proceeds through coupled adsorption–diffusion processes in which hydroxyl-rich fibrils create transient hydrogen-bond-mediated sorption sites that facilitate water migration across the membrane [2,26]. Consequently, moisture behavior depends on pore interconnectivity and fibrillar surface accessibility rather than exclusively on pore volume.

The maximum WVT observed in BP (400 ± 100 g m^−2^ day^−1^) is consistent with its open, low-density, interconnected fibrillar matrix identified by SEM, which imposes minimal tortuosity on vapor diffusion pathways. This aligns with reports showing that highly fibrillated, non-derivatized cellulose networks display higher permeabilities than their acetylated or more compact counterparts [33,34]. This is consistent with its open, low-density, interconnected fibrillar matrix identified by SEM, which imposes minimal tortuosity on vapor diffusion pathways. This aligns with reports showing that highly fibrillated, non-derivatized cellulose networks display higher permeabilities than their acetylated or more compact counterparts [2,3,20]. However, the lower moisture retention of BP (145.3 ± 12.3%) reduced the duration of water availability under hydric stress, explaining why superior transport did not translate into the highest germination rate.

In contrast, SB presented intermediate WVT values (300 ± 100 g m^−2^ day^−1^) combined with the highest moisture retention (215.7 ± 15.8%), reflecting an architecture that balances capillary stabilization with moderate vapor exchange [3,20]. This balance prolonged water residence at the seed–membrane interface while maintaining sufficient gaseous permeability, driving the highest germination rate (90.5 ± 0.99%), thereby confirming that germination performance under hydric stress is governed by the interplay between water delivery rate and water residence time rather than by a single transport parameter.

RH exhibited the lowest WVT (170 ± 60 g m^−2^ day^−1^), water absorption (186.4 ± 10.2%), and retention capacity (92.1 ± 6.5%), attributable to the compact, silica-reinforced architecture that restricts vapor flux through increased network tortuosity and reduced free volume [32]. Notably, this WVT value is comparable to that reported for a chemically hydrophobized lignin-cellulose nanofiber coating (158 ± 11 g m^−2^ day^−1^) [35,36], demonstrating that structural densification through biogenic silica retention can restrict vapor transport as effectively as chemical hydrophobization, a finding with direct implications for designing barrier performance through biomass selection rather than chemical modification.

### 3.4. Functional Performance in Germination and Early Growth

**Figure 4:**
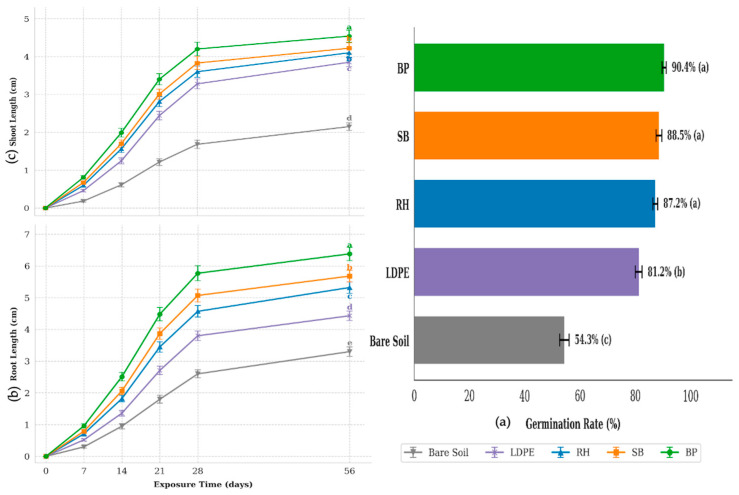
Germination and growth performance under water stress conditions. Boxplots of (**a**) germination percentage (%), (**b**) shoot length (cm), and (**c**) root length (cm) of *Brassica oleracea* var. *botrytis* grown under RH, SB, and BP membranes. Values represent mean distribution (*n* = 5). Different letters indicate significant differences (*p* < 0.05).

The biological validation confirmed that porous architecture and moisture regulation capacity significantly influence germination and seedling vigor under water stress (Table 2, Figure 4). SB membranes achieved the highest final germination rate (90.5 ^a^ ± 0.99%), statistically outperforming BP (87.0 ^b^ ± 0.76%) and RH (86.5 ^b^ ± 0.89%) (*p* < 0.05). All three cellulose membranes outperformed the bare soil negative control (Bare Soil: 54.3 ^c^ ± 2.10%), demonstrating a measurable improvement in germination under water-limited conditions attributable to membrane-mediated moisture retention. Germination rates under the cellulose membranes were superior to those recorded under the LDPE positive control (81.2 ^b^ ± 1.50%), confirming that the bio-based membranes are functionally competitive.

Growth kinetics recorded from day 0 to day 56 revealed distinct shoot and root development patterns depending on the membrane source (Table 2). At day 7, RH membranes showed the highest shoot length (2.5 ± 0.05 cm), whereas BP and SB exhibited lower initial elongation (1.5 ± 0.02 and 1.6 ± 0.04 cm, respectively). By day 28, SB seedlings surpassed the other treatments (8.2 ± 0.02 cm), a trend that was maintained at day 56, when SB reached the greatest final shoot length (9.7 ± 0.05 cm), followed by BP (8.7 ± 0.02 cm) and RH (7.3 ± 0.08 cm). All differences at day 56 were statistically significant (*p* < 0.05).

Root length at day 56 was significantly greater in BP (5.30 ± 0.01 cm) and RH (5.20 ± 0.03 cm) than in SB (4.40 ± 0.02 cm, *p* < 0.05). Root elongation under all biocomposite membranes substantially exceeded that observed in bare soil (3.3 ± 0.18 cm), confirming a positive biomechanical effect of membrane-mediated moisture availability on long-term root development under hydric stress. Under conventional LDPE mulch (4.4 ± 0.14 cm), root elongation was significantly lower than in the BP and RH treatments and statistically equivalent to SB. This behavior clarifies the distinct dual-phase mechanism governing these materials: while the rapid structural breakdown and high-water retention of SB maximize early seed germination, its premature degradation limits long-term soil moisture conditioning. Conversely, the enhanced root development under BP and RH membranes at day 56 is consistent with their prolonged structural integrity and distinct moisture-delivery profiles. The stable water vapor transport (WVT) of the BP matrix ensures a continuous, localized moisture flux that supports steady root elongation without triggering rapid material matrix decay. Meanwhile, the compact fibrillar arrangement and higher structural persistence of RH promote a targeted hydrotropic response; by maintaining a moderate, controlled moisture gradient near the surface, it stimulates deeper and more vigorous root exploration as the trial progresses.

Mechanical behavior and biological response emerged from the same underlying microstructural organization, demonstrating that stiffness, deformability, and plant performance are structurally coupled rather than independently determined properties. This coupling operates through hydrogen-bond organization and fibrillar arrangement, which simultaneously define load-bearing capacity and moisture-responsive behavior.

**Figure 5:**
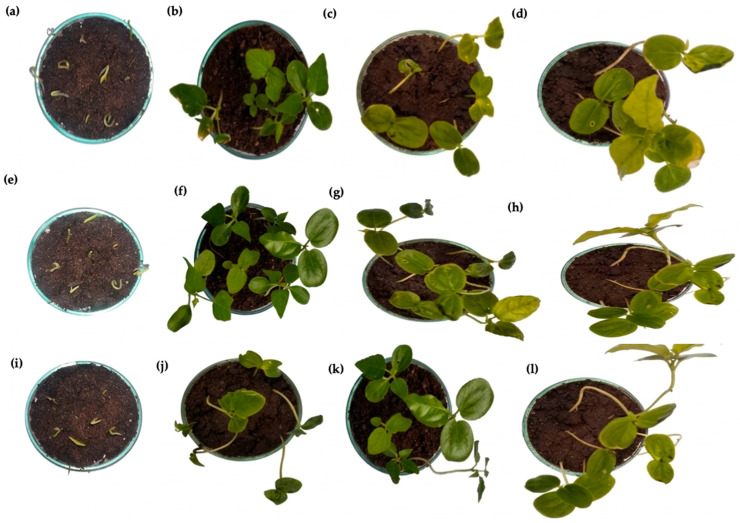
Seed germination and early growth under cellulose membranes. Photographic sequence of germination and early plant development under water stress conditions for (**a**–**d**) sugarcane bagasse (SB), (**e**–**h**) rice husk (RH), and (**i**–**l**) banana pseudostem (BP) at different exposure times: 0, 7, 14, 28, and 56 days, respectively.

The elevated compressive strength of RH (8.16 ± 0.24 MPa) originated from silica-mediated fibrillar confinement and restricted chain rearrangement, as mineral domains promote rigid load-bearing networks by limiting polymer mobility and enhancing local stress transfer efficiency [19,23]. However, this structural rigidity was associated with the lowest elongation (2.33 ± 0.17%) and reduced moisture transport, which may have contributed to the comparatively lower shoot elongation observed in RH seedlings (7.30 ± 0.08 cm). The physical restriction imposed by a dense, low-permeability matrix may increase mechanical resistance during radicle protrusion and limit local moisture redistribution around the emerging root system (Figure 5a–d).

In contrast, BP generated more compliant membranes (elongation: 9.83 ± 0.25%; compressive strength: 2.81 ± 0.15 MPa) through accessible hydrogen-bond interactions and greater fibrillar mobility. This structural compliance may reduce mechanical impedance during germination, facilitating radicle emergence and promoting rapid shoot elongation (Figure 5e–h), consistent with the highest shoot development recorded for BP seedlings (8.70 ± 0.02 cm) [10]. The favorable vapor transport of BP further supports continuous oxygen exchange and gas diffusion during early seedling development.

Notably, superior shoot growth under SB membranes (9.70 ± 0.05 cm) at the final evaluation (day 56) demonstrates that optimal long-term biological performance does not exclusively arise from maximum transport capacity or minimum structural rigidity. Instead, sustained plant development under water-stress conditions appears to depend on a sustained balance between capillary moisture stabilization, moderate vapor permeability, and adequate structural support conditions collectively provided by the intermediate porous architecture of SB (Figure 5i–l). This supports the concept that seed–matrix interactions are governed by the integrated coupling of transport and mechanical properties, rather than by either property in isolation, and underlines the importance of biomass-specific microstructural design for agricultural applications under hydric stress.

### 3.5. Biodegradation Behavior and Kinetics

**Figure 6:**
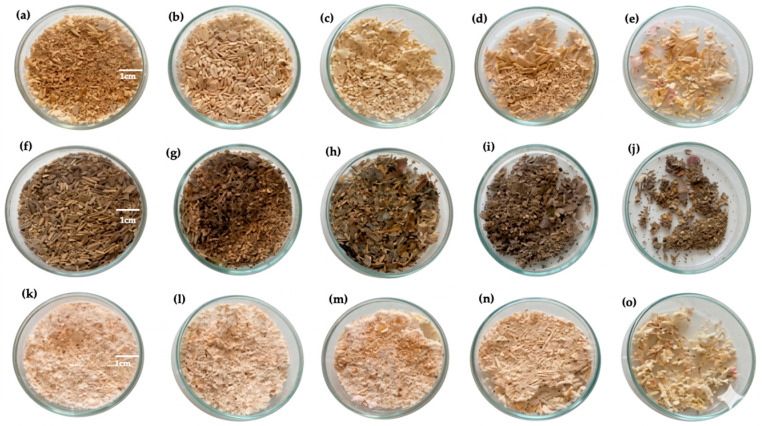
Macroscopic biodegradation behavior of cellulose membranes under soil conditions. Representative images showing degradation of (**a**–**e**) SB, (**f**–**j**) RH, and (**k**–**o**) BP membranes after 0, 7, 14, 28, and 56 days of soil burial. Progressive fragmentation and color changes indicate structural breakdown and decomposition.

**Figure 7:**
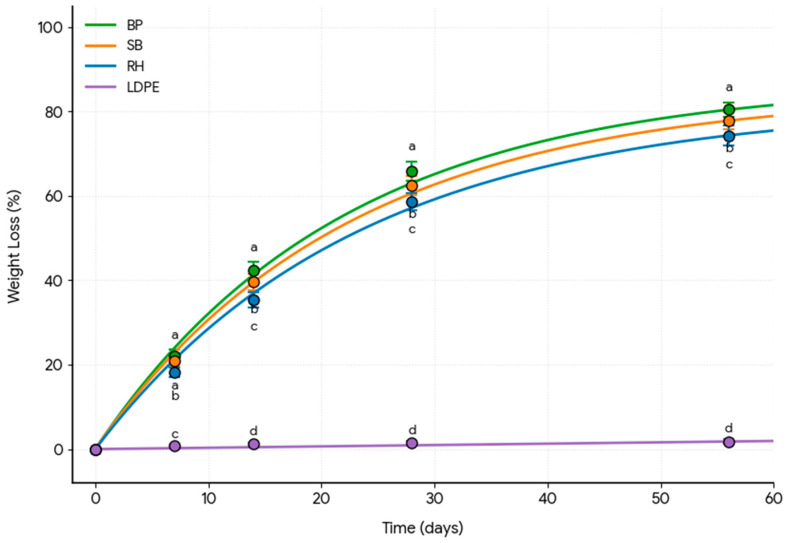
Biodegradation kinetics of porous cellulose membranes. Weight loss (%) of rice husk (RH), sugarcane bagasse (SB), and banana pseudostem (BP) membranes during 56-day soil burial degradation. Experimental data were fitted using a first-order exponential kinetic model. Values are presented as mean ± standard deviation (*n* = 5). Different lowercase letters indicate statistically significant differences among membranes at each sampling interval according to Tukey’s HSD test (*p* < 0.05).

All membranes underwent progressive mass loss over the 56-day soil burial period, accompanied by macroscopic fragmentation, loss of structural integrity, and surface darkening (Figure 6). Mass loss curves were well described by first-order exponential kinetics for all treatments (R^2^ ≥ 0.995), and degradation parameters differed among biomass sources (Table 3, Figure 7).

BP membranes exhibited the highest degradation rate constant (k = 0.046 day^−1^) and the shortest half-life (t_50_ = 15.0 days), with a model-predicted maximum mass loss of 80.4% at the plateau. SB membranes showed intermediate behavior (k = 0.045 day^−1^; t_50_ = 15.4 days; plateau: 87.1%), while RH membranes exhibited the lowest rate constant (k = 0.043 day^−1^), the longest half-life (t_50_ = 16.1 days), and the highest predicted maximum mass loss (96.3%). Although differences in k values were statistically significant (*p* < 0.05), their absolute magnitude was small (range: 0.003 day^−1^). The more substantive differentiation among membranes was observed in the plateau values, which ranged from 80.4% (BP) to 96.3% (RH), indicating biomass-dependent differences in the extent of long-term degradation rather than in the initial degradation rate.

Macroscopic degradation profiles further reflected these differences. BP membranes showed rapid fragmentation and loss of cohesion from day 14 onward, with near-complete structural disintegration observed by day 56. RH membranes maintained greater physical integrity during the early burial stages, with progressive fragmentation becoming evident after day 28. SB membranes exhibited transitional behavior, preserving partial structural cohesion during the first two weeks before undergoing progressive fragmentation through the remainder of the evaluation period (Figure 6).

Biodegradation behavior further reflected the molecular accessibility of each cellulose matrix, as microbial degradation of cellulose proceeds through water-assisted enzymatic hydrolysis requiring diffusion into accessible amorphous regions and exposure of β-(1 → 4)-glycosidic bonds to cellulolytic enzymes [25,27,31]. Consequently, degradation kinetics were conditioned by pore architecture and fibrillar organization in a pattern consistent with the structural hierarchy established.

BP exhibited the highest degradation rate constant (k = 0.046 day^−1^) and shortest half-life (t_50_ = 15.0 days), consistent with its interconnected porous structure and lower fibrillar packing density, which likely facilitated early microbial colonization, water penetration, and enzymatic surface exposure. Comparable relationships between open porosity, fibrillar accessibility, and accelerated biodegradation have been described in biodegradable agricultural films and cellulose-derived scaffolds [24,25]. The maximum predicted mass loss for BP (80.4% at 56 days, as derived from the fitted first-order exponential model Y = 80.4(1 − e^−0.046t^)) indicates extensive but not complete degradation within the experimental window, suggesting that residual structural fractions may persist beyond the observation period. This behavior is agronomically relevant, as it implies that BP membranes would substantially decompose within a single growing season without requiring mechanical removal.

RH exhibited the lowest degradation rate (k = 0.043 day^−1^; t_50_ = 16.1 days) and the highest maximum predicted mass loss (96.3%), the latter reflecting the eventual accessibility of cellulosic fractions after silica-domain disintegration. The initial persistence of RH is consistent with reports of mineral-mediated protection in silica-containing biopolymers, where inorganic domains restrict water penetration and limit enzymatic access during early degradation stages [19,25]. SB exhibited intermediate kinetics (k = 0.045 day^−1^; t_50_ = 15.4 days), supporting the interpretation that biodegradation rate is governed by the interaction between pore accessibility, moisture diffusion capacity, and fibrillar organization rather than by cellulose chemistry alone.

It should be noted that while the differences in k values among membranes (range: 0.003 day^−1^) are statistically significant at *p* < 0.05 (Tukey HSD), their practical magnitude is modest, suggesting that all three biomass sources produce membranes with broadly comparable degradation timelines under the soil conditions evaluated. The more meaningful differentiation lies in the predicted plateau values (96.3% for RH vs. 80.4% for BP), which reflect long-term degradation completeness and are more relevant for assessing soil residue accumulation in agricultural contexts. From an environmental perspective, this tunability in both degradation rate and long-term mass loss supports the strategic use of biomass selection as a design tool for tailoring membrane persistence to match specific crop establishment periods, reinforcing the circular bioeconomy potential of cellulose-based Nature-based Solutions for semi-arid agriculture [8,10,25].

## 4. Conclusions

Biomass origin determined the hydrogen-bond organization and porous architecture of the fabricated cellulose membranes, producing distinct mechanical, transport, and biological performance profiles. Critically, all three cellulose membranes significantly outperformed the conventional LDPE mulch control in seed germination, demonstrating that hydrogen-bond-mediated moisture regulation can surpass passive physical barrier exclusion as a strategy for agricultural water management. RH generated compact, silica-reinforced membranes with the highest structural resistance; BP produced the most open and permeable network with superior vapor transport and shoot development; and SB achieved the optimal balance between moisture retention and germination performance, confirming that sustained water availability at the seed surface is more determinant than maximum transport rate under hydric stress. Biodegradation kinetics followed first-order behavior for all membranes (R^2^ ≥ 0.995), with BP showing the fastest mass loss (plateau: 80.4% at 56 days) and RH the greatest long-term degradation extent (96.3%), demonstrating that membrane persistence is tunable through biomass selection. These findings provide a rational basis for designing biomass-specific cellulose membranes as biodegradable Nature-based Solutions for moisture regulation in water-limited agricultural systems. Future work should incorporate X-ray diffraction characterization, multi-species germination trials, and field-scale validation under semi-arid conditions to consolidate the agronomic applicability of these materials.

## Figures and Tables

**Table 1:** Physical, mechanical, and barrier properties of porous cellulose membranes.

Membranes	RH Rice Husk	BP Banana Pseudostem	SB Sugarcane Bagasse
Thickness (µm)	95.2 ± 0.5	95.7 ± 0.8	94.1 ± 1.5
Density (g cm^−3^)	1.33 ± 0.02	1.15 ± 0.06	0.89 ± 0.10
Weight (g)	0.32 ± 0.01	0.30 ± 0.01	0.35 ± 0.01
Water absorption (%)	186.4 ± 10.2	311.8 ± 18.6	485.3 ± 22.4
Water retention (%)	92.1 ± 6.5	145.3 ± 12.3	215.7 ± 15.8
Compressive Strength (MPa)	8.16 ^a^ ± 0.24	2.81 ^c^ ± 0.15	5.39 ^b^ ± 0.31
Elongation (%)	2.33 ^c^ ± 0.17	9.83 ^a^ ± 0.25	5.65 ^b^ ± 0.47
Water vapor Transmittance (g m^−2^ day^−1^)	170 ^c^ ± 60	400 ^a^ ± 100	300 ^b^ ± 100

Values are expressed as mean ± standard deviation (*n* = 5). Different lowercase letters within the same row indicate statistically significant differences among membrane sources (Tukey HSD, *p* < 0.05).

**Table 2:** Germination and growth parameters of *Brassica oleracea* var. *botrytis* cultivated under cellulose membranes. (**A**) Final germination performance. (**B**) Growth kinetics.

(**A**)					
**Membranes**	**RH**	**BP**	**SB**	**LDPE**	**Bare Soil**
Germination (%)	86.5 ^b^ ± 0.89	87.0 ^b^ ± 0.76	90.5 ^a^ ± 0.99	81.2 ^b^ ± 1.50	54.3 ^c^ ± 2.10
(**B**)					
**Day**	**Membranes**	**Shoot Length (cm)**	**Root Length (cm)**		
0	RH	0.0 ^a^ ± 0.00	0.0 ^a^ ± 0.00		
	SB	0.0 ^a^ ± 0.00	0.0 ^a^ ± 0.00		
	BP	0.0 ^a^ ± 0.00	0.0 ^a^ ± 0.00		
	LDPE	0.0 ^a^ ± 0.00	0.0 ^a^ ± 0.00		
	Bare Soil	0.0 ^a^ ± 0.00	0.0 ^a^ ± 0.00		
7	RH	2.5 ^a^ ± 0.05	0.7 ^b^ ± 0.02		
	SB	1.6 ^b^ ± 0.04	0.5 ^c^ ± 0.01		
	BP	1.5 ^b^ ± 0.02	0.9 ^a^ ± 0.02		
	LDPE	0.4 ^c^ ± 0.05	0.5 ^a^ ± 0.04		
	Bare Soil	0.2 ^c^ ± 0.04	0.3 ^b^ ± 0.05		
14	RH	4.6 ^a^ ± 0.09	1.6 ^b^ ± 0.01		
	SB	3.5 ^b^ ± 0.01	1.9 ^a^ ± 0.03		
	BP	4.4 ^a^ ± 0.07	1.5 ^b^ ± 0.04		
	LDPE	1.2 ^a^ ± 0.10	1.3 ^a^ ± 0.11		
	Bare Soil	0.6 ^b^ ± 0.07	0.9 ^b^ ± 0.10		
28	RH	6.1 ^b^ ± 0.02	3.2 ^b^ ± 0.01		
	SB	8.2 ^a^ ± 0.02	2.8 ^c^ ± 0.01		
	BP	6.5 ^b^ ± 0.10	3.6 ^a^ ± 0.02		
	LDPE	3.2 ^c^ ± 0.16	3.8 ^a^ ± 0.18		
	Bare Soil	1.6 ^d^ ± 0.14	2.6 ^b^ ± 0.15		
56	RH	7.3 ^c^ ± 0.08	5.2 ^a^ ± 0.03		
	SB	9.7 ^b^ ± 0.05	4.4 ^b^ ± 0.02		
	BP	8.7 ^a^ ± 0.02	5.3 ^a^ ± 0.01		
	LDPE	3.8 ^a^ ± 0.02	4.4 ^a^ ± 0.14		
	Bare Soil	2.1 ^b^ ± 0.12	3.3 ^b^ ± 0.18		

Final germination percentage was determined on day 7 following the ISTA (International Seed Testing Association) evaluation criteria for Brassica species, whereas shoot and root growth responses were monitored from day 0 to day 56. Values are presented as mean ± standard deviation (*n* = 5). Different lowercase letters indicate significant differences (*p* < 0.05).

**Table 3:** Kinetic parameters of cellulose membrane biodegradation.

Membranes	Regression Equation	R^2^	k (Day^−1^)	t_50_ (Days)
RH	$Y=96.3(1-e^{-0.043t})$	0.998	0.043	16.1
SB	$Y=87.1(1-e^{-0.045t})$	0.997	0.045	15.4
BP	$Y=80.4(1-e^{-0.046t})$	0.995	0.046	15.0
LDPE	$Y=2.0(1-e^{-0.001t})$	0.988	<0.001	>500

Degradation data were fitted using a first-order exponential model. k represents the degradation rate constant and t_50_ corresponds to degradation half-life.

## Data Availability

The data presented in this study are available upon request from the corresponding author.
